# Severe Acute Malnutrition: The Potential of Non-Peanut, Non-Milk Ready-to-Use Therapeutic Foods

**DOI:** 10.1007/s13668-023-00505-9

**Published:** 2023-10-28

**Authors:** Oluwaseun F. Akinmoladun, Oluwaseun P. Bamidele, Victoria A. Jideani, Cebisa N. Nesamvuni

**Affiliations:** 1 Department of Nutrition, Faculty of Health Sciences, University of Venda, Private Bag X5050, Thohoyandou, 0950 South Africa; 2 Department of Nutrition and Dietetics, College of Agriculture, Food Science and Technology, Wesley University, PMB 507 Ondo, Ondo State, Nigeria; 3 Department of Health Science, University of the People, Pasadena, CA 91101 USA; 4 Department of Food Science and Technology, University of Venda, Private Bag X5050, Thohoyandou, 09590 South Africa; 5 Department of Food Technology, Cape Peninsula University of Technology, P.O. Box 652, Cape Town, 8000 South Africa

**Keywords:** Severe acute malnutrition, Ready-to-use therapeutic foods, Alternative RUTFs, Effectiveness

## Abstract

**Purpose of Review:**

This review provides information on the prospect and effectiveness of ready-to-use therapeutic foods (RUTFs) produced locally without the addition of milk and peanut.

**Recent Findings:**

The foods used in fighting malnutrition in the past decades contributed little to the success of the alleviation program due to their non-effectiveness. Hence, RUTFs are introduced to fight malnutrition. The peanut allergies, the high cost of milk, and the high production cost of peanut RUTF have made its distribution, treatment spread, and accessibility very slow, especially in areas where it is highly needed. There is a need, therefore, for a low-cost RUTF that is acceptable and effective in treating severe acute malnutrition among under-5 children.

**Summary:**

This review shows both the success and failure of reported studies on the use of non-peanut and non-milk RUTF, including their cost of production as compared to the standard milk and peanut-based RUTF. It was hypothesised that replacing the milk ingredient component with legumes like soybeans can reduce the cost of production of RUTFs while also delivering an effective product in managing and treating severe acute malnutrition (SAM). Consumers generally accept them better because of their familiarity with the raw materials.

## Introduction

Based on cross-sectional surveys on the prevalence of stunting, over a million deaths are accountable for stunting [1] and [2] linked undernutrition to 45% of deaths among children below the age of 5 years. In Africa, 20% of infants die before their fifth birthday, while mortality affects 25.9% and 25% of children with stunted growth in South Africa and Uganda [3–5]. Aside from the mortality rate, the damaging effect of stunting is severe, and it is usually associated with intellectual impairment, severe infections, obesity, heart diseases, and diabetes in adolescence and adulthood [6–9]. It may be irreversible in under-5 children. The underlying cause of this malnutrition primarily results from inadequate dietary intake, inadequate breastfeeding, unemployment, and food insecurity induced by the high rate of income poverty [10]. According to the joint reports of UNICEF, WHO, IBRD, and WB, 149.2 million (22.0%), 45.4 million (6.7%), and 38.9 million (5.7%) of under-5 children globally were with stunted growth, wasted in weight, and overweight in 2020, respectively [2]. Unfortunately, Asia and Africa had the highest burden of this malnutrition [2]. In the document by [2], 53% and 41%, 70% and 27%, and 48% and 27% of these children were reported to be with stunted growth, wasted weight, and overweight in Asia and Africa, respectively. Among children with stunting, 23.3% were found in Southern Africa [2]. Although there was a reduction in children with stunted growth, in Southern Africa (29.1 to 23.3%) from 2000 to 2020, stunting in African children increased from 54.4 to 61.4 million from 2000 to 2020. Severe acute malnutrition (SAM) is a major public health concern due to its long-term health repercussions and high mortality.

The foods commonly used as approach to fight malnutrition in the last years, mainly enriched cereals and legumes, and flour-based foodstuffs enhanced with legumes and cereals with or without sugar, oil, milk, and eggs contributed little to the success of the interventions due to their non-effectiveness in SAM treatment [11–14] and some significant complications such as flatulence. Moreover, clean and safe water is scarce in most rural areas in sub-Sahara Africa, and contaminated water for cooking food is mostly the norm. Also, populations in these areas have high food spoilage due to microbial increase from high geographical temperatures [15]. Hence, the use and adoption of ready-to-use therapeutic food (RUTF) have proven successful in the last few years.

RUTF has been a solution to this crisis in the last few decades [15]. Most RUTF consists of peanuts augmented with powdered milk, vitamins, vegetable oil, sugar, and mineral salts. RUTF’s peculiarities are a complete nutritional composition with amino acids, vitamins, mineral salts, essential fatty acids, high energy density (between 500 and 540 kcal/100g), and an extended shelf-life (low moisture content). These RUTF products can be appropriately used on-site during therapeutic programmes. For instance, it can be used at home without going to nutritional rehabilitation centres or hospitals [15].

Production of peanut-based ready-to-use therapeutic food (P-RUTF) has helped reduce this severe malnutrition in developing countries [16, 17]. Despite the significant progress made in combating SAM, the distribution and treatment spread of the peanut-based ready-to-use therapeutic foods (RUTFs) in severe acute malnourishment (SAM) affected areas is slow due to the high cost of production [18]. About 10% of the world’s children with stunted growth have access to the RUTF treatments [18]. 

Food assistance programmes led by United Nations (UN) used RUTF as a vital component in treating SAM in low- and middle-income countries amongst children aged 24–59 months in regions affected by food security. Although RUTFs promised to be a potential solution to the treatment of SAM, 80% of children with SAM had no access to RUTF [19]. Based on the report of [18], the number of children with stunted growth should be lowered by 65 million and save 3.7 million lives if nutrition intervention reaches them by 2025. There are two significant reasons why RUTFs were not adequately addressing SAM. These are accessibility (affordability) and availability. On average, peanut RUTF costs $41–51(USD) per child. The high cost of shipping, manufacturing and ingredients are major obstacles to increasing the availability of RUTFs [20••].

Milk’s high-quality protein, mineral profile, high lactose and bioactive properties (anticancer, satiating, antimicrobial, hypotensive, anti-inflammatory, antioxidant and muscle and insulinotropic protein synthesis) incorporate milk as major raw material in RUTF production [21]. Hence, > 50% of the protein in RUTF should be from animal sources as specified by UN guidelines. In contrast to this affirmation statement, [22] reported that soya could substitute cow milk, given the sociocultural or medical discrepancies associated with cow milk in infants. Also, [23] declared that the nutritional profile of RUTF can be achieved without adding milk. A non-milk soya-maise-sorghum RUTF (SMS-RUTF) is as effective as P-RUTF in terms of weight gain, recovery rate and length of stay in the hospital in SAM treatment among children aged ≥ 24 months [24, 25]. This implies that P-RUTF, which is more expensive, should be used for children aged < 24 months, while SMS-RUTF should be used for children aged ≥ 24 months, which will reduce the cost of community-based management of acute malnutrition (CMAM) programmes. Considering the number of children living with malnutrition to be reached and treated, coupled with the low available budget for SAM treatment, there is a need for low-cost, effective alternative RUTFs. These RUTFs will not include peanut, milk and whey proteins in their ingredients and will be locally sourced for and manufactured [26]. This review aims to provide information on the potentials and efficacies of locally produced RUTFs without peanuts and the addition of milk as a significant ingredient in treating SAM in children. In addition, the successes and failures of documented alternative RUTFs in the past few decades used in treating SAM were also explored.

## Methodology

Grey literature and peer-review papers were scanned on the internet using the following keywords: ready-to-use therapeutic foods, efficacy, effectiveness, United Nations Children’s Fund (UNICEF) or World Health Organization (WHO). Google Scholar, Scopus and PubMed were used to search the literature. The search excluded any literature focusing on digestibility, molecular composition and policy dimensions of RUTF.

### The Need for Alternative RUTF

RUTF is an energy-dense, mostly paste, lipid-based that needs no cooking and can easily resist bacterial contamination, a significant component in the CMAM [27]. In the production of RUTF, all ingredients are ground into < 200-µm particle size. The carbohydrate and protein components are embedded in a lipid matrix with little or no water [27, 28]. With a low water activity product, there is resistance against contamination by bacteria which grants safely stored RUTF at ambient temperatures [27]. P-RUTF is the most widely used RUTF. It constitutes milk powder, peanut butter, vegetable oil, sugar, vitamin and minerals [17, 27, 28], and it is like the WHO’s F-100 milk nutritionally [29, 30]. [31, 32•] reported several countries where RUTF has been successfully used in CMAM to treat children with SAM in poor resource settings. It has shown low fatality cases and high recovery rates with more significant weight gain.

Since 2015, UNICEF has requested manufacturers to propose products based on using alternative ingredients for review and future consideration, including non-peanut-based components or alternatives to milk. Not only can alternative ingredients generate cost savings in producing RUTF, but non-peanut recipes also increase acceptability in many countries where peanut-based products are not popular. Some alternative RUTFs use different legumes and cereals instead of peanuts (soy, chickpea flour, lentils or oats). They have a similar texture as paste and comply with the compositional guidance of the 2007 Joint statement, and most can use the existing machinery in RUTF manufacturing facilities [33].

Although there is high effectiveness in the management of SAM with P-RUTF, its cost of production is expensive. In 2009, a metric ton of non-peanut RUTF costs $1583, while peanut RUTF costs $2393 [25]. There is a limitation in processing these RUTFs in the needed countries due to the unavailability of locally produced milk powder and awkwardness in the available peanuts meeting the UN standards from aflatoxin levels [25]. Hence, there is a barrier in the procurement and production of RUTF in the needed countries, which led to the innovation of a new RUTF that uses locally available and acceptable materials for processing.

A substantial fraction of the children who suffered malnutrition came from the poorest parts of every country. However, most of these countries do not get enough support from non-governmental organisations (NGOs) to fight widespread acute malnutrition. There is, therefore, a need for cost-efficient and effective solutions to reduce malnutrition to achieve sustainable development goals as RUTF has changed the treatment of SAM as an excellent alternative to in-patient treatment [34]. Sadly, RUTFs were available to only 15% of those children in need of it worldwide [35]. In 2007, UNICEF used *Plumpy’Nut* (peanut-based) as the only RUTF for treating SAM in children [36]. Although the standard RUTF is potent in many cases, the challenges of its acceptability, cost and availability prevent it from reaching the target population. These caused researchers to study ways to improve acceptability amongst the target populace and reduce production costs. Based on research, food-assisted products obtained with locally sourced ingredients overcome the high cost and acceptability issues common with standard RUTFs [37]. Consequently, the potential of locally sourced and acquired RUTFs has motivated researchers to erupt alternative RUTFs that are cost-saving, acceptable by the recipients and efficient in treating SAM.

Many RUTFs that do not use peanut and milk protein have been trialled to increase local production, improve local acceptance and reduce cost. These formulations include egg powder and insect proteins [33]. In Colombia, UNICEF developed a fish-based wafer snack locally to treat SAM. Its production cost is 20% cheaper than the standard RUTF formulation, with good taste acceptability results among children affected with SAM [38]. A novel RUTF called *Nutreal* is nutritionally like *Plumpy’Nut* and made locally with the ingredients from the target population’s diet like pulses and cereals. There was a positive acceptability result, effective in SAM’s treatment among local consumers and reduced production cost than peanut-based RUTF not locally produced [39].

The formulation of alternative RUTFs led to the techniques modified to the target population’s local conditions and tastes [20••]. Based on efficacy studies, there is a broad acceptance of locally produced RUTFs among the recipients; hence, the alternative RUTFs can get to malnourished and at-risk children. In Bangladesh, RUTF made using rice, lentils and chickpea demonstrated positive and promising results [40]. Also, there were positive outcomes in the body composition and anthropometric indices of children treated with milk and soy-based RUTF [41•]. It was observed that both [40, 41•] improved on the past research on the efficacy of locally produced RUTF in the treatment of malnourished children in Malawi [17]. Additionally, [26] reported affordability and effectiveness in treating SAM using locally formulated chickpeas RUTFs in Ethiopia. Also, locally available RUTF produced from sorghum and millet showed efficacy in SAM treatment in Tanzania [42]. [43] reviewed that standard and novel RUTFs made little or no difference in recovery among children with SAM, and the effect of standard RUTF on relapse and mortality compared to the alternative novel RUTFs is unknown.

Furthermore, processing, animal source foods and fortification improve the protein quality of plant-based foods to meet protein requirements [44]. Based on [45], soybeans can replace animal products in infant formula and produce a favourable amino acid profile. Moreover, substituting soybeans with milk in the production of RUTF may enlarge its availability to malnourished children and reduce the cost of production; hence, this offers an alternative for RUTF production without or with little milk as raw materials [41•]. According to [45], standard milk-based formula (M-RUTF) containing 25% milk was more effective than whole soy flour-RUTF with 10% milk in treating kwashiorkor children. However, the undehulled soy used by [45] had less bioavailability, more anti-nutrients and lower amino acid digestibility. Such a nutrient profile implies that the body might not utilise the available nutrients for recovery. Alternatively, dehulling increases digestibility and lowers anti-nutrient availability in crops. Hence, processing methods like fermentation that will increase the bioavailability of nutrients should be implored during RUTF production.

Uhiara and Onwuka [46] used okra seeds extract to replace milk in RUTF production. The extract was used to feed rats for 3 weeks. The overall acceptability of the okra seeds extract-RUTF by fifteen-man semi-trained panellists showed no significant difference compared to the standard RUTF. Also, [47] replace milk with *Locusta migratoria* as the protein source in producing RUTFs. This locust-RUTF had tannin and phytate below the allowable limits in RUTFs, and other nutritional parameters met the requirements of WHO on RUTF; hence, it can be used as an alternative to milk in the production of RUTF.

### Various Forms of RUTF

RUTFs appear in different forms, which include liquid, semi-solid and solid. Although RUTFs come in other forms, the semi-solid in the form of paste is the most popular form of RUTF. The liquid is usually informed of drinkable foods for easy swallowing among malnourished children. This was produced by [48] in Uganda. [49] produced a powder RUTF drink called *Mushpro*. This was made from mushroom, wheat flour, skimmed milk powder and cocoa powder. Also, [50] and [51] produced biscuits using mung bean flour and soybean flour.

### The Potential and Effectiveness of Non-Peanut–Based RUTFs

Valid Nutrition (a research firm) used locally grown crops such as soya, maise and sorghum to produce peanut-free and milk-free SMS-RUTF. SMS-RUTF increases the prospect of using locally grown ingredients and reduces production costs. The efficacy of SMS-RUTF had been compared with P-RUTF. Although the effectiveness of SMS-RUTF was assessed by [25], the result was inconclusive. The SMS and P-RUTF were below the international standard with a high level of mortality. This inclusive result could be because of the measles and cholera outbreak during the study period. However, [42] improved the composition of SMS-RUTF using the results of [25]. These improvements included enrichment of SMS-RUTF with crystalline amino acids that led to high phytic acid (PA) and zinc molar ratio, PA and iron molar ratio and enhanced omega-6/omega-3 fatty acid profile ratio. Hence, the efficacy, haemoglobin, amino acid profile, weight gain, length of hospital stay, rate of recovery and participant body composition using SMS-RUTF were determined and compared with P-RUTF [42]. Phytic acid that binds divalent metallic ions and affects their absorption in the small intestine is absent in animal foods but present in plants. The SMS-RUTF used de-germinated maise, dehulled soybean and specially made mineral and vitamin premixes. The finished product met the recommended RUTF vitamin and mineral levels by WHO [29]. The iron and zinc levels were raised above the recommended concentration to compensate for high PA in the SMS-RUTF. Also, vitamin C content was increased to increase iron bioavailability in the product. Content of n-3 PUFA was increased while n-6 was decreased. In the production of RUTFs, [52] showed that the high level of phytates in legumes and cereals does not stop their use as raw materials to produce RUTF. Hence, there was an increase in vitamin C and an iron level of their formulated RUTF to achieve optimum iron absorption through increased vitamin C/iron weight ratio and phytic acid/iron molar ratio. The intended outcome was a boost in SAM survivors’ child development, resulting from reduced anaemia and improved iron status, which was linked to the inhibitory effect that milk protein has on iron absorption.

Milk-free-soya-maize-sorghum (FSMS-RUTF) and a 9.3% milk-soya-maise-sorghum (MSMS-RUTF) were compared to amino acid (AA) concentration of PM-RUTF in SAM children aged 6–59 months [53, 54]. Both milk-free (FSMS-RUTF) and 9.3% milk (MSMS-RUTF) showed no inferiority to the plasma leucine, methionine, cysteine and EAA concentration of PM-RUTF at discharge in children aged 6–59 months [42, 53]. FSMS-RUTF and MSMS-RUTF supplied proteins and amino acids like PM-RUTF for adequate recovery from SAM. Alternatively, [55] used unprocessed soy flour to treat SAM and observed a lower growth rate. Unprocessed soy flour contains anti-nutrients responsible for lower growth and recovery rate with 10% milk RUTF. The study was a clinical quasi-effectiveness trial and not a strict efficacy trial. Therefore, the lower growth and recovery rate obtained by [55] might result from anti-nutrients in the hull of soy. In a study conducted by [56], produced alternative-RUTF (A-RUTF), sorghum and soybean flour replaced 50% of peanut while non-fat dried milk and whey protein concentrate gave 50% of the protein. [56] reported A-RUTF to be inferior to standard-RUTF (S-RUTF) in the management of SAM due to lower mid-upper arm circumference (MUAC) and weight gain; hence, A-RUTF did not facilitate recovery in SAM children.

The efficacy study of [42] on SMS-RUTF in Malawi checked the intention to treat (ITT), length of stay in the hospital (LOS) and recovery rate as compared to P-RUTF. ITT analysis met minimum international standards in the SMS-RUTF produced for children aged 24–59 months but contradicted 6–23 months. Lower recovery rate, higher mortality, defaulter and non-response rate were observed by [42] for SMS-RUTF in children aged 6–23 months, but the recovery rate was not inferior to the P-RUTF in children aged 24–59 months. Also, SMS-RUTF was not inferior to P-RUTF in weight gain, LOS and anaemia, while SMS-RUTF gives higher haemoglobin. Energy intake was higher in P-RUTF than SMS-RUTF among the two age groups. SMS-RUTF caused flatulence to a few children aged < 24 months and a higher report of dislike and side effects of SMS-RUTF among defaulted children [42]. Methionine, proline and tyrosine were lower in SMS-RUTF than in P-RUTF which may contribute to the inferiority of SMS-RUTF among children aged < 24 months [42]. Also, there was a reduction in the bioavailability of iron and zinc in the SMS-RUTF. However, the shift from milk to plant source could cause an increase in the phytic acid with a decrease in the bioavailability of zinc and iron.

### Cost-Effectiveness of RUTFs

Although standard RUTF requires at least 50% of its protein from products of high protein quality like milk, the high cost of milk has increased the cost of production. Formulation of alternative for standard RUTF with lower or no milk is necessary for production scale-up and local availability. There is a need to assess the cost-effectiveness of no-milk or lower milk RUTF to comprehend if they could be allowed broader coverage in SAM treatment. In a study by [57], milk was replaced with soybeans to provide protein in the formulation of RUTFs. The results showed high effective products in the treatment of SAM, and their cost of production was cheaper than *phumphy’nut.* Alternative formulations of RUTF could potentially be optimised with micronutrients and possibly be made less expensive using other ingredients instead of skim milk. Table 1 summarises the potential of alternative ready-to-use therapeutic foods in managing acute malnutrition.

**Table 1:** Summary of studies on the potential of alternative ready-to-use therapeutic foods in managing acute malnutrition

Constituents of RUTF	SS	Study design	Parameters tested	Result	Conclusion	References
Replaces milk with soybeans						
Soya, maise and sorghum	1927	Non-blind, parallel, cluster-randomised equivalent trial	Recovery rate, Adjusted risk difference (ARD)	SMS-RUTF has a lower recovery rate than P-RUTF	The result was inconclusive in their equality with recovery rates	[25]
	225	Non-blinded 3 arm, parallel-group, simple randomised control trials	LOS, haemoglobin, iron store	FSMS-RUTF was not inferior to PM-RUTF in recovery rates in both groups. FSMS-RUTF was superior in haemoglobin and body iron stored recovery	FSMS-RUTF is more effective in treating and remedying iron deficiency anaemia in severe acute malnourished children	[42]
	389	Simple randomised controlled non-inferiority trials	Anaemia, Iron status, Retinol-binding protein, Gut inflammation	SMS-RUTF increased body iron stores while P-RUTF had the highest recovery rate	SMS-RUTF is more potent in correcting BIS and treating anaemia than P-RUTF	[52];
Soy-based with soy protein isolate	260	Randomised double-blind intervention trial	Acceptability, weight and MUAC gain, TBW, fat	The two samples showed similar taste acceptability trial improvements in anthropometric indices, body composition, total body water, fat-free mass and fat mass	There is a need for a multi-centre study to generalise the findings	[41•]
Rice, mung beans and soy	200	Randomised crossover trials/design	Acceptability trial, weight and BMI (children and adults)	69% and 91% of HEBI and 65% and 81% of Plumpy’Nut were consumed by children and adults, respectively. There were statistical differences in weight and BMI gain between adults who consumed RUTF and the control	Both HIV-positive children and adults preferred and accepted HEBI. The weight gain translated significantly in BMI but not in MUAC	[89]
Unprocessed soy flour	1874	Randomised double-blind, clinical trial	Weight and height gain, recovery rate	Weight gain, height gain and recovery rate were higher in children receiving 25% milk RUTF than 10% milk RUTF	10% milk RUTF is less effective than 25% milk RUTF in the treatment of SAM	[55]
Milk-free and reduced milk	499	Non-blinded, 3-arm, parallel-group simple randomised control trial	Plasma amino acid, anthropometric measurements	FSMS-RUTF and MSMS-RUTF were not less in plasma leucine, methionine, cystine and EAA concentration in 6–59 months SAM children at discharge	There were sufficient functional metabolites of plasma amino acids by the three RUTF, a non-inferior in plasma methionine and cysteine in both FSMS-RUTF and MSMS-RUTF	[53, 54]
Fish and insects replace milk						
Fish-based	121	A single-blinded randomised controlled trial	Weight gain, anthropometry measurement	No statistical difference in weight gain and anthropometry measurement between the two groups	None of the RUTF was superior to the other and can serve as an alternative to milk based RUTF	[38]
Other pulses and cereal used in replacing peanut						
Chickpeas, soy and maise	2425	Cluster-randomised controlled trial	The recovery rate for MAM children, Cost-effectiveness	Super Cereal PLUS children showed a higher overall recovery rate. Children less than 24 months recovered more than those older than 24 months	Formula 2 is the most cost-effective of the four samples	[26]
Crystalline AA, Zn and vitamin C	1650	A non-blinded, parallel-group, simple randomised controlled trial	Changes in haemoglobin, PAA profile, Recovery rate, Weight gain, LOS, BIA	Both products met the minimum international standard for the 24–59 months group on ITT. SMS-RUTF was not inferior to P-RUTF in both groups’ weight gain and LOS. There was no difference in FM, FMI and BIA among the groups and control at discharge. P-RUTF was more consumed	Both SMS-RUTF and P-RUTF replenished all AA except methionine. SMS-RUTF was not inferior to P-RUTF in ≥ 24 months with recovery rate, LOS and weight gain. SMS-RUTF can correct anaemia	[90]
Whey protein concentrate	600	Blinded, randomise, clinical trial	Average weight gain, recovery rate, and LOS	WPC-RUTF was not inferior to P-RUTF	WPC34 is cheaper and more effective than P-RUTF. Also, the same processes are used in the production of both RUTF	[24]
Reduced quantity	802	Non-random sampling	Body composition, FFMI, FFM, FM	FFM, FMI and FM were similar in both reducing and standard RUTF but a higher FFMI and a lower Z. There was an incomplete FM recovery. There was no effect of a reduced dose of RUTF on the body composition of children by recovery	FFM was the highest weight gain during SAM treatment. A reduced dose of RUTF does not affect tissue creation compared to standard dose treatment	[91]

Fat-free mass index (FFMI), fat-free mass (FFM), fat mass (FM); length of stay (LOS), plasma amino acid profile (PAA), bioelectric impedance analysis (BIA), amino acid (AA), sample size (SS)

Ready-to-use supplementary foods (RUSF) are widely used in the cure of moderately acute malnutrition (MAM) [16, 58]. [26] reported the cost-effectiveness of four different RUSF (chickpea only, chickpea-maise-soya, super cereal (SC) and super cereal plus) used in treating MAM in Ethiopia. SC produced the required nutritional value at minimal cost in this report with no statistical significance among other products except super cereal plus and was well accepted by most of the targeted populace in the community. The ingredients that were used were procured, and production was local. [13] reported SC as a favourite meal for complementary and supplementary feeding programs for malnourished lactating and pregnant women, HIV and AIDS patients and under-5 children. Nevertheless, [12] reported SC as nutritionally inadequate and inappropriate in terms of energy density (0.5 kcal/g instead of the 0.8 kcal/g) that was recommended. Hence, even with the improved micronutrient profile, SC is ineffective in treating MAM among children of 6–23 months. However, the high amount of fibre and anti-nutrients reduced mineral absorption. Also, SC flour needs to be cooked before consumption, leading to extra costs from cooking utensils, fuel, and safe water. These inadequacies led to adding vegetable oil to meet energy and essential fatty acids requirements. Even though SC was the favourite meal among their studied population, [13] reported a lower recovery rate of 67% from malnutrition using SC than the 75% recovery rate standard, the minimum standard in response to a disaster. Females in all four products (chickpea only, chickpea-maise-soya, SC and SC+) with an exemption of Formular 1(chickpea only) have a lower recovery rate than males. In contrast, 6–11 months age group children had the highest recovery rate except for chickpea-maise-soya treatment. SC+ gave the highest recovery rate at ages 6–11 months. As a result of the sub-standard development in not being able to meet up with the standard recovery rate above, Super Cereal PLUS (SC+) was developed.

**Table 2:** Macronutrient contents of some locally produced RUTF

	Moisture (%)	Protein (%)	Fat (%)	Ash (%)	Crude fibre (%)	Carbohydrate (%)	Total energy(kcal/100 g)	References
Phumpy’nut	NR	13.6	35.7	NR	NR	43.48	545	[15]
*Metu2*	9.8	11.8	35.1	1.4	1.2	40.6	528.0	[83]
Chickpea RUTF (100%)	5.33	12.87	24.41	2.04	1.97	NR	NR	[88]
Mung bean RUTF (100%)	3.00	13.74	23.21	2.29	1.76	NR	NR	[88]
AOB	2.73	22.7	43.04	3.5	NR	19.67(g)	555	[67]
BOC	0.63	24.11	45.11	4.38	NR	17.83(g)	573	[67]
PCO	0.59	21.70	32.14	2.92	NR	36.73(g)	523	[67]
RUTF (25% milk)	NR	15(g/100 g)	40(g/100 g)	NR	NR	NR	2000(KJ/100 g)	[55]
RUTF (10%)	NR	15(g/100 g)	40(g/100 g)	NR	NR	NR	2000(KJ/100 g)	[55]
SMS-RUTF	NR	13.6 g	30.5 g	NR	NR	55.0 g	503.5	[92]
Novel RUTF	2.07	12.42	31.33	3.51	2.17	NR	530	[84]
Product 1	4.98	14.47	19.05	5.27	9.32	11.64	1137.65(KJ)	[57]
Product 2	7.65	15.43	18.00	7.74	9.51	39.75	1584.62(kj)	[57]
Product 3	6.80	12.30	19.21	4.24	8.65	23.67	1308.69(kj)	[57]
L-RUTF/(92 g)	NR	14	33	NR	NR	53	565(Kcal)	[93••]

Product 1: 30% soybean, 25% peanut, 15% soy oil, 28% sugar, 2% mineral mix. Product 2: 55% soybean, 28% maize flour, 15% soy oil, 2% mineral mix. Product 3: 30% soybean, 28% maize flour, 15% soy oil, 25% sugar, 2% mineral mix

*NR *Not reported*, AOB* peanut, date, and soybean, *BOC* acha, soybean, and cashew nut, Crayfish, *PCO* peanut, guinea corn, and soybean

SC+ has milk to meet children’s micronutrient requirement of 6–23 months. Hence, SC+ met the WHO requirement for MAM treatment due to the improved micronutrient premix, sugar, oil and milk powder [28]. Although SC+ has the same premix as SC, the sugar, oil and milk content differ. SC+ has a higher energy density of 0.7 kcal/g than 0.5 kcal/g in SC when prepared.

**Table 3:** Micronutrients contents of some locally produced RUTF

	K (mg/100 g)	Mg (mg/100 g)	Zn	Fe	Cu	Na	Vit A μg/100 g	Ca	Ref
*Phumphy’nut*	1111	92	14	11.5	1.78	189	o.91(mg)	320	[15]
*Metu2*	410.95	114.32	1.70	5.53	0.38	101.05	0.52	Nil	[83]
Chickpea RUTF (100%)	875.0	189.0	3.40	6.10	NR	24.0	NR	128.0	[88]
L-RUTF	1111	92	14	11.5	1.8	< 267	0.91(mg)	320(mg)	[93••]
Mung bean RUTF (100%)	1246.0	176.4	3.08	5.80	NR	15.0	NR	134.0	[88]
RUTF (25% milk)	1111	92	14	11.53	1.78	NR	910	NR	[55]
RUTF (10%)	1110	92	14	11.50	1.74	NR	913	NR	[55]
SMS-RUTF	NR	NR	18.7	52.4	NR	NR	NR	NR	[92]
CS-RUTF	935.6	NR	12.4	10.5	1.7	NR	816.9	304.1	[94]

Product 1: 30% soybean, 25% peanut, 15% soy oil, 28% sugar, 2% mineral mix. Product 2: 55% soybean, 28% maize flour, 15% soy oil, 2% mineral mix. Product 3: 30% soybean, 28% maize flour, 15% soy oil, 25% sugar, 2% mineral mix

*NR *Not reported*, AOB* peanut, date, and soybean, *BOC* acha, soybean, and cashew nut, Crayfish, *PCO* peanut, guinea corn, and soybean

### Cost of Dietary Treatment for Malnutrition

SAM and stunting are accountable for 21% of disability-adjusted life years (DALY) for under-5 children [1, 59]. To reduce the death rate in under-5 children with severe acute malnutrition, [60] examined the cost-effectiveness of community-based management of acute malnutrition (CMAM). The decision tree model juxtaposes the cost and effectiveness of SAM treatment in the health services with and without CMAM in Malawi. [60] and [61] observed that for the base case (both children that survived or died in the CMAM programme), the cost of CMAM was US$42 and US$26 per DALY averted, while the worst-case (account for children that stayed and died in CMAM programme) scenario accounted for US$493 and US$335 per DALY. Based on the definition of WHO, CMAM was highly cost-effective in the base case because the cost per DALY falls under Malawi’s gross national income (GNI) per capita of US$250 [29, 60]. This was also within the scope of DALY for other child health interventions. In Bangladesh, [62] reported US$1344 and US$ 26 per DALY averted for in-patient treatment and community-based strategy cost, respectively, which result in the cost of community treatment of SAM being one-sixth of that of in-patient treatment. Also, CMAM was still cost-effective even in the worst case in Malawi and Bangladesh [60, 62]. [61] reported 15,016 DALYs to be averted with $23 per DALY averted estimated cost using community-based treatment and prevention programmes for SAM in slums in India. The disability burden and death associated with SAM may be more significant when considering other adverse effects like nutritional oedema.

The cost of community-based therapeutic care (CTC) accounts for US$53 per DALY gained, US$1760 per life saved and a mean cost of US$203 per child [63]. Based on the above report, 36%, 13%, 17% and 34% of the total cost were from RUTF used, health centre visits, hospital admissions and technical support, respectively, during programme establishment. Seventy per cent (70%) of the cost of an average CTC per child was from the price of RUFT and one life saved for every 8.7 children that received CTC [63].

Screening and treatment of different levels of acute malnutrition in under-five children in a community setting can be achieved through community-based management of acute malnutrition (CMAM) [64]. Acute malnutrition can either be SAM or MAM. SAM relates to an 11-fold escalation mortality risk, and MAM is associated with threefold mortality risk. Hence, the cost of dietary treatment of SAM and MAM differs.

- Cost of dietary treatment of SAM
  The high cost of milk is a significant barrier to using milk-based RUTF [65]. About half of the expenses for SAM treatment are on therapeutic foods, and greater than 50% of these therapeutic foods are solely from milk powder [17, 66]. Based on Oakley et al.’s report, 10% milk with 15% soya RUTF used in the treatment of SAM had a slower recovery rate, weight and height gain than those receiving 25% milk. For the cost of milk-based RUTF production to be reduced, the content of skimmed milk was replaced with whey protein concentrate [56]. Sosanya et al. confirmed the lower cost of production in the locally produced RUTFs against the commercially produced RUTFs. [68] evaluated the cost-effectiveness of two CTC and therapeutic feeding centre (TFC) programmes in treating SAM. The mean cost per under-5 child treated with CTC and TFC was $134.88 and $284.56, respectively, while institutional cost per child treatment in CTC and TFC was $128.58 and $262.62, respectively. This higher institutional price could be connected to the fact that 43.2% of the institutional price of CTC went to RUTF. The authors concluded that CTC was more cost-effective than TFC. However, local production of RUTF can reduce the cost of RUTF, reducing the cost of CTC per child.
- Cost of dietary treatment of MAM
  SAM and MAM children have 11 and 3 times more likely to die than their non-malnourished counterparts [69]. Knowing fully well that if MAM is not well treated or controlled, it will lead to a more life-threatening condition like SAM. MAM management should be considered a public health issue. Based on the number of MAM children to be reached with RUSF, the cost of production of RUSF is an essential factor considering the global budget. Moreover, due to the high price of peanut and whey proteins, there is a need for alternative RUSF specifically for MAM children [70, 71]. Hence, chickpea-based RUSFs were improved [26].

According to [72], the ingredients approach was used to identify and analyse the cost of all resources used in the community treatment of MAM. The price includes the cost of screening, infrastructure and recurrent expenditure (supplementary food, personnel, materials, and medical supplies) per child. They used the trial document review and interviews with the key informant to estimate the cost. In the sum treatment of MAM based on [72] report, supplementary foods, personnel, materials and medical supplies cost 28–45%, 22–30% and 7–10% treatment cost, respectively.

The economic cost of the CMAM programme was estimated by [73] using a cost analysis design with a retrospective cross-sectional study in Ghana. A semi-structured questionnaire was used to collect information from under-5 children’s caregivers on household cost data. In contrast, interviews with crucial health personnel and reviewed documents were used to obtain the programme cost data. In [73] report, the programme’s economic cost was approximated to be $27,633.5, which refresher training made up 34% of the total cost. The financial household cost was approximately $1905.32 ($47.63 per household), with 79% direct cost. To treat one SAM case based on the report of [73] was $805.36 when using the CMAM protocol in Ghana.

### Comparisons of Anti-nutritional Factors in RUTF Made from Different Local Foods Sources

The natural form of stored phosphate in plants is phytic acid. This phytate formed indigestible and insoluble complexes that inhibit minerals’ bioavailability. The use of added or intrinsic phytase enzyme to reduce phytic acid and the use of iron in a chelated form such as sodium iron ethylenediaminetetraacetate (EDTA) reduce the impeding effect of phytic acid [74]. Mineral bioavailability can be estimated by phytate and mineral molar ratio in improving iron absorption in legume and cereal-based foods [74]. Negative zinc balance can result from a phytate/zinc ratio equal to or greater than 15, significantly lowering zinc absorption [75, 76].

The ability of condensed tannins to precipitate gelatine, alkaloids and other proteins makes them anti-nutrients [77, 78]. The most available plant polyphenols are condensed tannins (proanthocyanidins and catechin). They are the polymers or oligomers of flavan-3-ols. Tannins in legume seeds contain anti-nutritional components that might prevent the utilisation of nutrients efficiently and interfere with digestion [79, 80]. Even though a high intake of tannins is associated with carcinogenesis, no evidence of toxicity on excess consumption of tannins in legumes has been reported. Instead, tannins have been reported to have antidiabetic and antioxidant properties [81]. Legumes’ protein becomes more digestible when trypsin inhibitors are deactivated. Trypsin inhibitors prevent protein digestion by contributing to the loss of chymotrypsin and trypsin in the gut [82]. Phytates, condensed tannins, and trypsin inhibitors were higher in metu2 (a locally made RUTF comprising peanut, honey, ghee and sorghum) than *Plumpy’Nut*. At the same time, aflatoxins in metu2 were lower than Plumpy’Nut and within acceptable limits [83]. [84] reduced the anti-nutritional factors in the chickpea before being used by roasting the chickpea in the sand at 250 °C with an internal temperature of 140 ± 10 °C for 10 min.

### Comparisons of Macronutrients and Micronutrients of Some Already Produced Non-Milk RUTF with P-RUTF

Traditional foods of plant origin used for readily acceptable and nutritious foods like RUTF in developing countries increase the consumption of high-quality foods. The major constituents of the locally produced RUTF include pulses, cereals, minerals, vitamins, flavouring and skim-milk powder [85]. Legumes are an essential source of protein for low-income families. According to [86], legumes should be included in preparing effective, low-cost infant foods. [84] analysed chickpea-based RUTF-fed rats and compared the results with standard casein and peanut-based RUTF (*Plumpy’Nut*). Based on [84] report, the proximate analysis of chickpeas was related to *Plumpy’nut*. According to [84], true digestibility, biological value and net protein utilisation for chickpeas, *Plumpy’Nut* and casein were 83.78, 86.98 and 93.16; 87.77, 89.01 and 92.98 and 73.54, 77.42 and 86.58, respectively. However, chickpea RUTF and *Plumpy’Nut* showed no significant difference in other proteins measured except in casein. The protein efficiency ratio differs among the samples, and chickpea RUTF has a more excellent feed efficiency ratio than *Plumpy’Nut*.

Locally produced RUTF comprises lipids, carbohydrates and proteins with low indigestible fibre, vitamins and minerals that classify them as energy-dense foods; hence, it can treat SAM [84]. [83] compared *metu2* with *Plumpy’Nut* in North-eastern Uganda and discovered that *metu2* had higher energy content than *plumpy'nut* (528 kcal/100 g to 509 kcal). The vitamins K and A of *metu2* were lower than the WHO recommendation for RUTF in SAM treatment. However, essential fatty acids, Mg and Na, meet SAM treatment and recovery requirements. Although the Zn content in the *phumpy’nut* was higher, it was recorded that both metu2 and *phumpy’nut* were below WHO recommendation. Peanut, date and soybean (AOB); acha, soybean, and cashew nut; crayfish (BOC) and peanut; guinea corn and soybean (PCO) were the three RUTFs produced by [67]. Their energy level was discovered to conform with the WHO energy recommendation of 520–550 kcal/100 g. Hence, these RUTFs (AOB, BOC and PCO) were apt for treating SAM in vulnerable individuals and under-5 children [67]. The protein content of the produced RUTFs was greater than that of *Plumpy’Nut* [67]. Also, the fat content of AOB and BOC was greater than the fat content of *Plumpy’Nut.* [84] reveal that Plumpy’Nut and locally produced RUTF possess the same digestibility. Both products showed no difference in their proximate composition, net protein utilisation (NPU), true digestibility (TD) and biological value (BV). It was concluded that the minimal presence of non-digestible fibres, vitamins and minerals makes the RUTF energy-dense and suitable for malnutrition treatments. The macro- and micro-nutrient contents of some locally produced RUTF are shown in Tables 2 and 3, respectively.

### Consumer Acceptability of Most Locally Produced RUTF

The senses of taste, touch, smell, and vision determine consumers’ acceptability of new products. Texture and appearance are two critical tests relating to the preferences shown by consumers. Using locally available raw materials to produce a new product has resulted in better acceptability by consumers due to their familiarity with the raw materials’ texture, taste and aroma [57, 87]. RUTF with 100% chickpea produced in Pakistan was preferred based on its smoothness, flavour, texture and overall acceptability due to the daily chickpea consumption among the Pakistans [88]. Also, RUTFs, made from soybean, maise and peanut, were highly acceptable by the consumers due to their familiarity with the taste of the raw materials [57]. The sensory properties scores of some locally produced RUTFs are shown in Table 4.

**Table 4:** Sensory properties scores of some locally produced RUTF

	Appearance/colour	Flavour/aroma	Texture/consistency	Mouthfeel/taste	Smoothness	Overall acceptability	References
Chickpea RUTF (100%)	7.89	7.96	7.48	7.68	7.69	7.78	[88]
Mung bean RUTF (100%)	6.16	6.12	5.80	6.16	5.68	5.88	[88]
AOB	3.56	3.5/5	NR	3.48	NR	NR	[67]
BOC	3.76	3.88/5	NR	3.88	NR	NR	[67]
PCO	3.50	3.68/5	NR	3.24	NR	NR	[67]
Product 1	6.50	6.50	6.10	NR	NR	NR	[57]
Product 2	7.94	5.08	6.58	NR	NR	NR	[57]
Product 3	6.90	6.40	5.82	NR	NR	NR	[57]
L-RUTF	8.0	8.1	7.9	7.9	NR	NR	[93••]

Product 1: 30% soybean, 25% peanut, 15% soy oil, 28% sugar, 2% mineral mix. Product 2: 55% soybean, 28% maize flour, 15% soy oil, 2% mineral mix. Product 3: 30% soybean, 28% maize flour, 15% soy oil, 25% sugar, 2% mineral mix

*NR *Not reported*, AOB* peanut, date, and soybean, *BOC* acha, soybean, and cashew nut, crayfish, *PCO* peanut, guinea corn, and soybean

## Conclusions

There are always means of improving the already produced RUTF for SAM treatment in all reviewed publications. These improvements include nutrient contents, formulations, effectiveness, cost of production and acceptability by the intending consumers. However, alternative RUTFs that can be efficacious, meet cost efficiency goals, accessible and acceptable to the recipients can be developed by researchers to meet the world demand for RUTF in treating SAM. This development has led to more research in developing novel RUTF using locally grown and available agricultural produce in different areas of the world that will efficiently manage SAM. Researchers are moving daily to achieve these goals by developing alternative RUTF using local agricultural products and reducing production costs.

The nutritional profiling in this review shows that the nutritional content of RUTF can be achieved without adding milk or using peanuts. This will reduce the cost of production of RUTF because milk stands as the most expensive raw material in the processing.

Also, processing methods used in the production of RUTF can affect the nutrient content of the final product. These processing methods may reduce the bioavailability of most nutrients and increase the anti-nutritional factors of the RUTFs. However, adding some enzymes can reduce the effects of these anti-nutrient factors.

This review showed that standard and novel RUTFs made little or no difference in recovery among children with SAM. The effect of standard RUTF on relapse and mortality compared to the alternative novel RUTFs is unknown. The above showed the need for a follow-up on the long-term outcome of alternative RUTFs in treating malnutrition.
